# Transcriptional and genetic characteristic of chimera pea generation via double ethyl methanesulfonate-induced mutation revealed by transcription analysis

**DOI:** 10.3389/fpls.2024.1439547

**Published:** 2024-10-01

**Authors:** Jinglei Hu, Mingxia Liu, Dongxia Wang, Yunlong Liang, Yuan Zong, Yun Li, Dong Cao, Baolong Liu

**Affiliations:** ^1^ Key Laboratory of Crop Molecular Breeding, Key Laboratory of Adaptation and Evolution of Plateau Biota, Northwest Institute of Plateau Biology, Chinese Academy of Sciences, Xining, Qinghai, China; ^2^ College of Life Sciences, University of Chinese Academy of Sciences, Beijing, China; ^3^ College of Agriculture and Animal Husbandry, Qinghai University, Xining, Qinghai, China

**Keywords:** *Pisum sativum*, chimera, EMS mutation, transcriptome, SNP

## Abstract

Ethyl methanesulfonate (EMS)-induced mutagenesis is a prominent method for generating plant mutants, often resulting in chimera plants; however, their transcriptional and genetic characteristic remain elusive. In this investigation, chimera pea (*Pisum sativum* L.) specimens, labeled GY1 and GY2, exhibiting a distinctive phenotype with yellow and green leaves were meticulously cultivated via sequential double EMS mutagenesis. The observed color disparity between the yellow and green leaves was attributed to a significant reduction in chlorophyll content coupled with heightened lutein levels in both chimeric variants. Transcriptome profiling revealed the enrichment of differentially expressed genes in both GY1 and GY2, specifically implicating Kyoto Encyclopedia of Genes and Genomes pathways linked to amino acid biosynthesis and ribosome development, alongside Gene Ontology (GO) biological processes linked with stress response mechanisms. Few structural genes associated with chlorophyll and lutein biosynthesis exhibited discernible differential expression. Despite these functional similarities, distinctive nuances were evident between specimens, with GY1 exhibiting enrichment in GO pathways related to chloroplast development and GY2 showing enrichment for ribosome development pathways. Single-nucleotide polymorphism (SNP) analysis uncovered a shared pool of 599 and 598 polymorphisms in the yellow and green leaves of GY1 and GY2, respectively, likely stemming from the initial EMS mutagenesis step. Further investigation revealed an increased number of unique SNPs in the yellow leaves following the second EMS application, whereas the green leaves exhibited sparse and unique SNP occurrences, suggestive of potential evasion from secondary mutagenesis. This inherent genetic variability underpins the mechanism underlying the formation of chimera plants. Predominant base mutations induced by EMS were characterized by G/A and C/T transitions, constituting 74.1% of the total mutations, aligning with established EMS mutation induction paradigms. Notably, genes encoding the eukaryotic translation initiation factor eIIso4G and the ubiquitin ligase RKP, known to modulate leaf color in model plants, harbored two SNPs in the yellow leaves of both GY1 and GY2, implicating their putative role in the yellow leaf phenotype. Collectively, this study provides novel insights into the transcriptional and genetic characteristics of chimera plants via EMS-induced mutagenesis.

## 1 Introduction

Plant leaves play a pivotal role in plant development, as they absorb light energy for photosynthesis, producing organic compounds vital for growth. The green hue of leaves is attributed to their richness in chlorophyll, a crucial component for photosynthesis housed within chloroplasts (Matile et al., 1999). Accordingly, leaf color serves as a reflection of chlorophyll content, and the brightness of the color correlates with photosynthetic efficiency (Zhu et al., 2024). Leaf color variation is a common natural phenomenon influenced by physiological processes such as chlorophyll metabolism, carotenoid metabolism, anthocyanin metabolism, chloroplast development, and ribosome development (Mou et al., 2015). Disruption in the function or expression of genes involved in these processes can lead to changes in leaf color, consequently affecting photosynthesis and impeding plant growth and development (Henry et al., 2016). Ornamental horticultural plants are typically bred to have colorful leaves to meet consumer preferences, prompting researchers to explore methods to induce leaf color changes for esthetic enhancement (Davis and Burns, 2016). Furthermore, mutations in leaf color provide an opportunity to uncover the genetic mechanisms governing these physiological processes.

Physical and chemical mutagenesis methods are frequently employed to induce mutations resulting in leaf color variations. One of the most common methods to induce mutagenesis in plants is ethyl methane sulfonate (EMS) treatment, which offers advantages such as higher point mutation rates, fewer chromosomal aberrations, and simpler mutant screening compared to alternative methods (Kong et al., 2020). This approach has been successfully utilized to generate mutant libraries in various crops, including wheat (Cheng et al., 2008), cantaloupe (Song et al., 2015), tomato (Liu et al., 2017), and cucumber (He, 2019), facilitating the elucidation of genetic mechanisms underlying essential traits. However, the genetic mechanisms underlying the formation of chimera plants, a common outcome of mutagenesis, remain poorly understood.

Peas (*Pisum sativum* L.) are annual or biennial climbing herbaceous plants that are commonly employed for genetic research owing to their adaptability to poor soil conditions and preference for cool, humid climates (Holland, 1919). Serving as an important model organism in genetics, peas have contributed significantly to the discovery of Mendelian genetic laws (Smýkal, 2014). Moreover, their relatively large leaves and continuous growth provide ample material for investigating chimera plants.

In this study, double EMS mutagenesis treatment was employed to generate mutant pea libraries, resulting in the identification of several chimeric plants with variegated leaves (Watanabe et al., 2007). Transcriptome analysis and bioinformatics were conducted to elucidate the transcriptional and genetic characteristics underlying the chimera phenotype.

## 2 Materials and methods

### 2.1 EMS mutagenesis and sampling

About 5000 seeds were selected from the experimental field of the Molecular Breeding Laboratory for Cereal Crops at the Northwest Plateau Institute of Biology, Chinese Academy of Sciences, from the pea cultivar GaoWan 1, were immersed in a 1% EMS solution for 12 hours, followed by rinsing with running water for 48 hours (Sega, 1984). Subsequently, these treated seeds were planted with conventional field management in Balang Village, Xining City, Qinghai Province (31°24′ 18.97′′ N, 121°29′ 21.8′′ E). After a growth period of four months, seeds from the M1 generation were harvested as a mixed population. These M1 generation seeds were then subjected to the same treatment protocol of immersion in a 1% EMS solution for 12 h, followed by rinsing with running water for 48 h, in accordance with the protocol outlined by Sega (1984). This resulted in the M2 generation seeds. The resulting seeds were then sown, yielding pea plants exhibiting chimerism characterized by leaves displaying a distinct pattern of half yellow and half green. We selected plants with more obvious phenotypic characteristics, such as plants with yellow leaves that are clearly expressed and can be clearly contrasted with green leaves. When plants have already shown this characteristic, they are marked for subsequent observation and sampling. The yellow and green leaves from these chimeric plants were individually harvested (Bolger et al., 2014)rapidly frozen in liquid nitrogen, and stored at –80°C for subsequent chemical compound analysis and RNA-sequencing.Yellow leaves in the chimera were labeled to GY1Y and GY2Y, and green leaves were labeled as GY1G, and GY2G.

### 2.2 Chlorophyll content measurement

The extraction and determination of chlorophyll were performed according to the national standard NY/T3082-2017. In brief, the fresh leaves were ground with liquid nitrogen and 0.25 g of the obtained powder was placed in a 15-mL centrifuge tube to which 2.5 mL of a 1:1 (V:V) mixture of anhydrous ethanol and acetone was immediately added, followed by vortexing for 10 s to mix well. The centrifuge tube was stored at 4°C overnight covered in tin foil to avoid light exposure and prevent evaporation. The following day, the tubes were centrifuged for 10 min and the chlorophyll content was determined by measuring the absorbance values of the supernatant at 470 nm, 645 nm, and 663 nm with an enzyme-linked immunosorbent assay reader (Thermo Fisher Scientific, China) (Palta, 1990); a 1:1 (V: V) mixture of anhydrous ethanol and acetone served as a blank solution for withering. The chlorophyll content was measured for each group with three biological replicates. The contents of chlorophyll a (chlA), chlorophyll b (chlB), and total chlorophyll were then calculated with the following formulas:


chlA content (mg/g)=[(12.72×A1)–(2.59×A2)]×V/(1000×m)

chlB content(mg/g)=[22.88×A2]–(4.76×A1)]×V/(1000∗m)

Total chlorophyll content(mg/g) =[(8.75×A1)+(20.29×A2)]×V/(1000×m)

Where A1 is the absorbance value of the supernatant at 663 nm, A2 is the absorbance value of the supernatant at 645 nm, V is the volume of the test solution in milliliters, and m is the sample mass in grams. The calculation result was retained with three significant digits.

### 2.3 Lutein content determination

A homogenous sample was accurately weighed to 1.35 ± 0.01 g and placed in a 50-mL polypropylene centrifuge tube. Ten milliliters of the extraction solvent, prepared by dissolving 1 g of BHT in 200 ml of cyclohexane and then adding 400 ml of ether and 400 ml of n-hexane, was added to the tube to prevent light exposure. The mixture was then vortexed for 3 minutes, followed by centrifugation at 4500 rpm for 3 min. This extraction process was repeated twice and the collected extracts were combined. The resulting solution was concentrated under reduced pressure at room temperature until nearly dry and then vortexed and dissolved in the extraction solvent. This operation was repeated once more, and the combined extraction solvents were thoroughly mixed and set aside for purification. The solution was passed through an activated neutral alumina solid-phase extraction cartridge at a flow rate of approximately 1 mL/min. Elution was carried out using 3 mL of extraction solvent, and the effluent and eluent were combined before concentration to near dryness under reduced pressure at room temperature. The residue was dissolved in a solution of butylated hydroxytoluene (BHT) in ethanol with the volume adjusted to 2 mL and subsequently passed through a 0.45-μm filter membrane for liquid chromatography-based measurement (Aruna et al., 2009) on an Agilent 1260 chromatograph with a JADE-PAK column (250 mm × 4.6 mm, 5 μm internal diameter) maintained at 30°C. The mobile phase consisted of methanol/water (88/12 volume ratio, containing 0.1% BHT) and methyl tert-butyl ether (containing 0.1% BHT). A gradient elution was performed from 0 to 18 min, during which the methanol/water ratio was decreased sequentially from 100% to 10%. At 18.1 min, the methanol/water ratio was increased from 10% to 100%, and this composition was maintained for 10 min.

### 2.4 RNA-sequencing

RNA was extracted from the specimens with the DP432 RNA extraction kit (Tiangen, China); the extraction was repeated three times per sample. The concentration of the extracted total RNA was measured with a NanoDrop microvolume spectrophotometer (Thermo Fisher Scientific, China) and the quality of the total RNA was detected with 1.0% agarose gel electrophoresis. The transcriptome sequencing was carried out by Parsenor Gene Technology Co., Ltd. on an Illumina HiSeq 2000 platform with a read length of 100 bp (Minoche et al., 2011).

### 2.5 Unigene function annotation and classification

After obtaining RNA-Seq sequencing data, Trimmomatic was used to trim low-quality reads and adapter sequences to obtain clean reads, and the clean reads was aligned to the reference genome using (Kim et al., 2019) (Bolger et al., 2014). The reference genome was CAAS Psat ZW6 1.0 (GCF_024323335.1). StringTie was used to assemble transcripts from the alignment results and quantify expression (Pertea et al., 2015). FeatureCounts was used to count reads using existing annotation files (Liao et al., 2013). Salmon was used for transcript quantitative analysis, and R package DESeq2 was used to identify genes that were differentially expressed (Patro, 2015). The clusterProfiler package was used to perform GO (GO; http://www.geneontology.org) and KEGG (KEGG; http://www.genome.jp/kegg/) functional enrichment analysis (Yu et al., 2012; Alexa and Rahnenführer, 2009). Finally, visual analysis was performed to generate volcano maps, heat maps, and principal component analysis (Abdi and Williams, 2010).

### 2.6 Single-nucleotide polymorphism (SNP) calling

Varscan (v2.3.9) software was employed for SNP detection (Koboldt et al., 2013). The filtering criteria applied were as follows: (1) base quality score (base Q) > 20, (2) minimum coverage of the SNP site by more than 8 reads; (3) at least 2 reads supporting the mutation site, and (4) P-value for the SNP site below 0.01.

## 3 Results

### 3.1 Chimeric pea plants generated by double mutagenesis (EMS treatment) procedure

**Figure 1:**
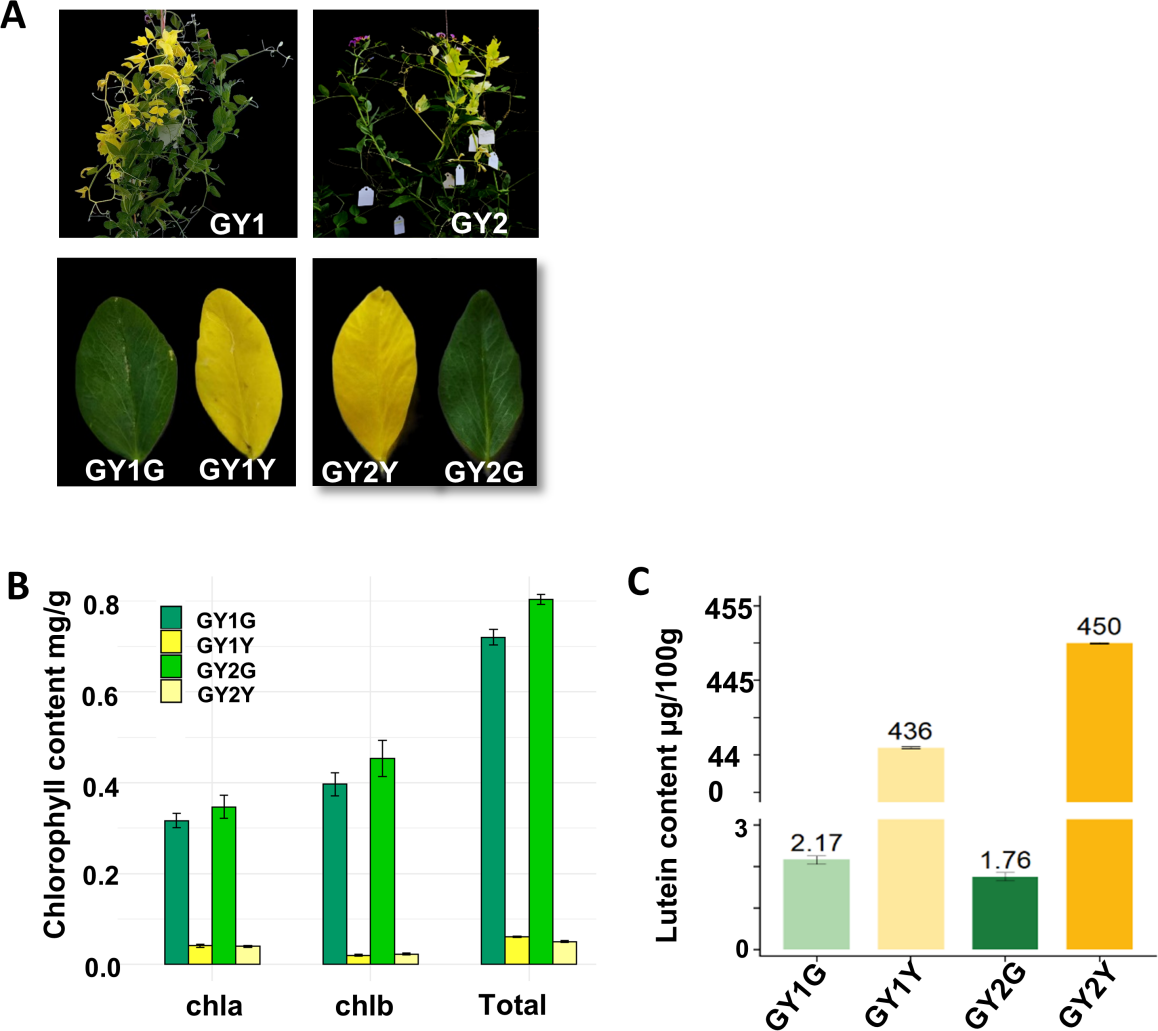
The phenotype of the chimeric plants, and the chlorophyll and lutein contents of their yellow and green leaves. **(A)** The phenotype of GY1 and GY2. GY1G represents the green leaves of GY1, GY1Y represents the yellow leaves of GY1, GY2G represents the green leaves of GY2, and GY2Y represents the yellow leaves of GY2. **(B)** Chlorophyll content of GY1 and GY2. **(C)** Lutein content of GY1 and GY2.

From the 2383 M1 lines obtained, a total of six chimeric pea plants were identified among 1245 M2 lines, all displaying partial white or yellow leaves. Among these, two chimeric lines (designated GY1 and GY2) with a distinct mixed yellow and green leaf phenotype were selected for further investigation (**Figure 1A**). Given the alteration in leaf color from green to yellow, it was anticipated that there would be corresponding changes in chlorophyll content. As expected, chlorophyll content analysis revealed a notable reduction in total chlorophyll, chlA, and chlB levels in the yellow leaves compared to those in the green leaves in both GY1 and GY2. Specifically, we conducted three biological replicates. The average value was taken. The total chlorophyll content in the yellow leaves of GY1 and GY2 was 0.06 mg/g and 0.05 mg/g, respectively. Chlorophyll a was 0.04, and chlorophyll b was 0.02. The total chlorophyll content in the green leaves reached 0.73 mg/g and 0.81 mg/g, respectively. Chlorophyll a was 0.3 and 0.35, respectively. Chlorophyll b was 0.42 and 0.46, respectively. These data fully demonstrate that in these two chimeric plants, the chlorophyll content of yellow leaves is much lower than that of green leaves (**Figure 1B**; **Supplementary Table S2**). Lutein content analysis further demonstrated higher levels of lutein in the yellow leaves compared to those in the green leaves. Specifically, the lutein content in yellow leaves was 436 μg/100g and 450 μg/100 g, whereas that in the green leaves was 2.17 μg/100 g and 1.76 μg/100 g in GY1 and GY2, respectively (**Figure 1C**; **Supplementary Table S3**). These quantitative disparities in both chlorophyll and lutein contents signified the chemical basis underlying the observed color changes.

### 3.2 Transcriptome differences between yellow and green leaves in chimeric pea lines

The observed differences in compound accumulation were considered to be likely attributed to variations in gene expression. We conducted transcriptome sequencing using the Illumina HiSeq 2000 platform, generating 41.1 Mb, 43.07 Mb, 40.37 Mb, and 52.28 Mb of raw reads for the yellow leaves of GY1 (GY1Y), yellow leaves of GY2 (GY2Y), green leaves of GY1 (GY1G), and green leaves of GY2 (GY2G), respectively. After filtering, we obtained 40.60 M, 42.41 M, 39.67 M, and 49.51 M clean reads with Q20 scores of 97.92%, 97.98%, 97.93%, and 97.85% for these same four lines, respectively, indicating high data reliability for further analysis (**Supplementary Table S3**).

**Figure 2:**
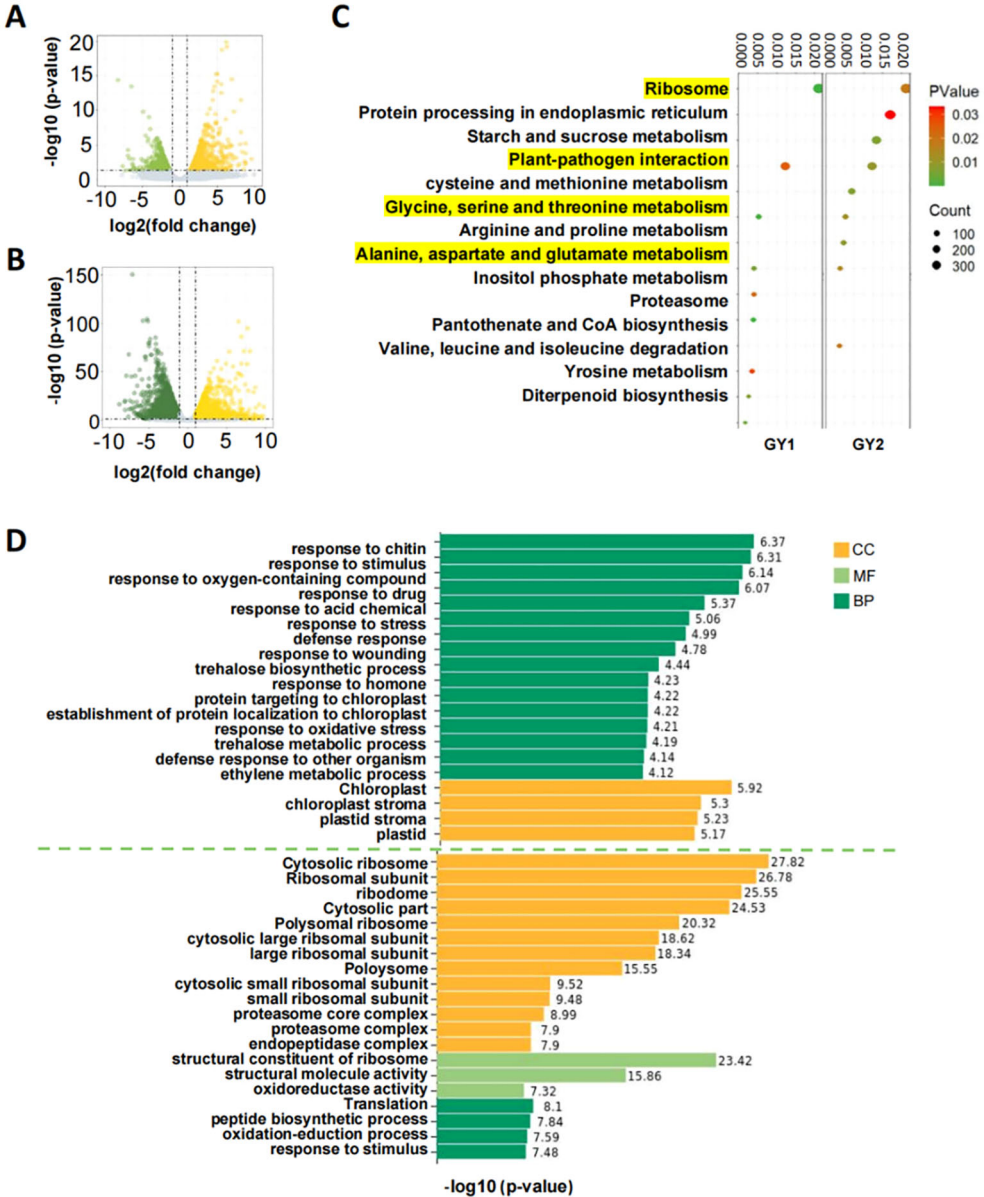
Volcano plot, KEGG enrichment plot, and GO enrichment histogram of differentially expressed genes. **(A)** Volcano plot of differentially expressed genes in the GY1 line; yellow represents up-regulated genes, which means the genes had higher expression in yellow leaves and green represents down-regulated genes, which means the genes had lower expression in yellow leaves. **(B)** Volcano plot of differentially expressed genes in the GY2 line; yellow represents up-regulated genes and green represents down-regulated genes. **(C)** KEGG enrichment factor diagram of differentially expressed genes in the GY1 and GY2 lines. The vertical axis is the KEGG metabolic pathway and the horizontal axis is the gene ratio. The size of the circle in the figure represents the total number of differentially expressed genes enriched in the pathway and the color of the circle represents their corresponding P values. The yellow highlighted part represents the common KEGG metabolic pathways of GY1 and GY2. **(D)** GO enrichment histogram of differentially expressed genes in the GY1 and GY2 lines. GY1 is above the green dotted line and GY2 is below the dotted line. CC, cellular component (yellow); MF, molecular function (light green); BP, biological process (dark green).

Inter-sample correlation analysis showed that the correlations between biological replicates exceeded 0.9, confirming the suitability of the data for further analysis (**Supplementary Figure S1**). DEGs were identified by comparing the FPKM values of yellow leaves to green leaves in GY1 and GY2. We found 1693 down-regulated and 1879 up-regulated genes in GY1, and we found 2984 up-regulated and 2615 down-regulated genes in GY2 (**Figures 2A, B**). GO and KEGG enrichment analyses were performed to elucidate the biological functions of these DEGs (**Figures 2C, D**). The KEGG pathways enriched in both GY1 and GY2 were primarily involved in amino acid anabolism and ribosome development, which can affect chlorophyll biosynthesis to varying degrees (**Figure 2C**). GO enrichment analysis revealed that DEGs in GY1 were enriched in chloroplast development, whereas those in GY2 were primarily enriched in ribosome development (**Figure 2D**). Other enriched pathways included amino acid pathways involving chlorophyll synthesis, such as the cysteine ​​metabolism pathway (**Supplementary Figure S2**).

**Figure 3:**
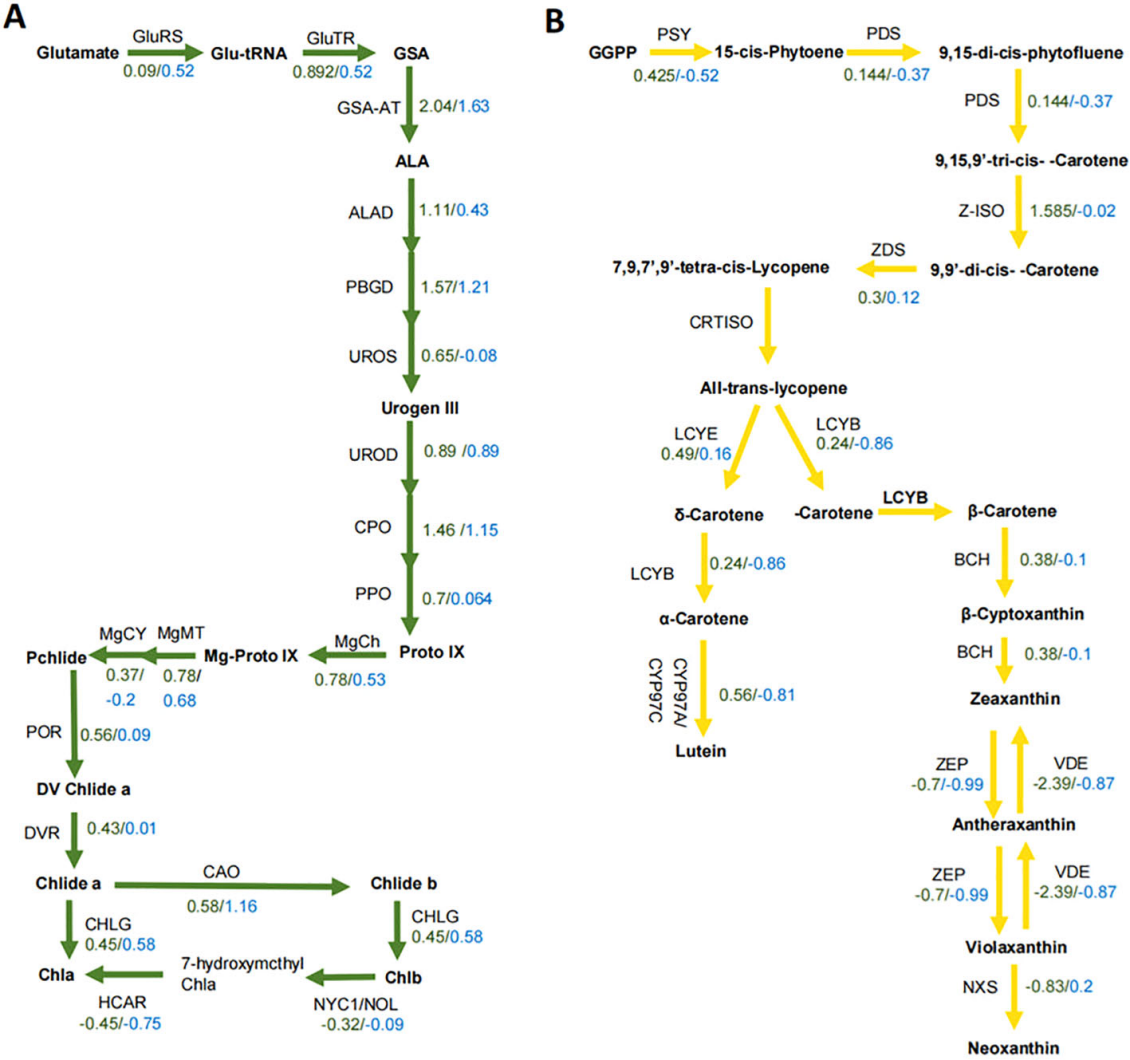
The expression of genes in the chlorophyll and lutein biosynthesis pathways in the GY1 and GY2 lines. **(A)** The expression levels of genes in the chlorophyll biosynthesis pathway for GY1 and GY2. Green numbers represent the log_2_ fold change value in GY1 and blue numbers represent the log_2_ fold change value in GY2. GluRS, Glu-tRNA synthetase; GluTR, Glu-tRNA reductase; GSA, Glu-1-semialdehyde; GSA-AT, Glu-1-semialdehyde aminotransferase; ALA, 5-aminoleculinic acid; ALAD, ALA dehydratase; PBGD, porphobilinogen deaminase; UROS, urogen III synthase; Urogen III, uroporphyrinogen III; UROD, urogen III decarboxylase; CPO, coprogen III oxidase; PPO, protoporphyrinogen IX oxidase; Proto IX, protoporphyrin IX; MgCh, magnesium chelatase; Mg-ProtoIX, Mg-protoporphyrin IX; MgMT, Mg-Proto IX methyltransferase; MgCY, Mg-Proto IX monomethyl ester cyclase; Pchlide, protochlorophyllide; POR, light-dependent NADPH, Pchlide oxidoreductase; DV Chlide a, 3,8-divinyl chlorophyllide a; DVR, D-vinyl reductase; Chlide a, chlorophyllide a; CAO, chlorophyllide a oxygenase; Chlide b, chlorophyllide b; CHLG, chlorophyll synthase; NYC1, non-yellow coloring; NOL, non-yellow coloring-like; HCAR, 7-hydroxymethyl Chl a reductase. **(B)** The expression of genes in the lutein biosynthesis pathway for GY1 and GY2. Green numbers represent the log_2_ fold change value in GY1 and blue numbers represent the log_2_ fold change value in GY2. GGPP, geranylgeranyl diphosphate; PSY, phytoene synthase; PDS, phytoene desaturase; Z-ISO, ζ-carotene isomerase; ZDS, ζ-carotene desaturase; CRTISO, carotene isomerase; LCYE, lycopene ϵ-cyclase; LCYB, lycopene β-cyclase; CYP, cytochrome P450 carotene hydroxylase; BCH, β-carotene hydrolase; ZEP, zeaxanthin epoxidase; VDE, violaxanthin deepoxidase; NXS, neoxanthin synthase.

Given the differences in chlorophyll and lutein contents between the yellow and green leaves, we selected genes involved in chlorophyll and lutein biosynthesis for comparative expression analysis. The log2 fold change of structural genes for chlorophyll biosynthesis ranged from –0.45 to 2.00 in GY1 and from –0.75 to 1.63 in GY2 (**Figure 3A**). For lutein biosynthesis, the log2 fold change ranged from –2.39 to 1.585 in GY1 and from –0.87 to 0.2 in GY2 (**Figure 3B**). Although the major genes related to chlorophyll and lutein biosynthesis were more highly expressed in the yellow leaves than in the green leaves, no single gene could be definitively linked to the differential accumulation of chlorophyll and lutein.

### 3.3 Function of mutations generated by double EMS-induced mutagenesis

A total of 56,359 valid SNP loci were identified by comparing the sequences to the pea reference genome. Among them, 54,535 SNPs showed the same genotypes in yellow and green leaves of GY1 and GY2. These SNPs likely represent the genetic differences between the QingWan 1 cultivar and the reference genome. Additionally, 599 and 598 SNPs were found in both the yellow and green leaves of GY1 and GY2, respectively, likely resulting from the first EMS mutagenesis treatment.

**Figure 4:**
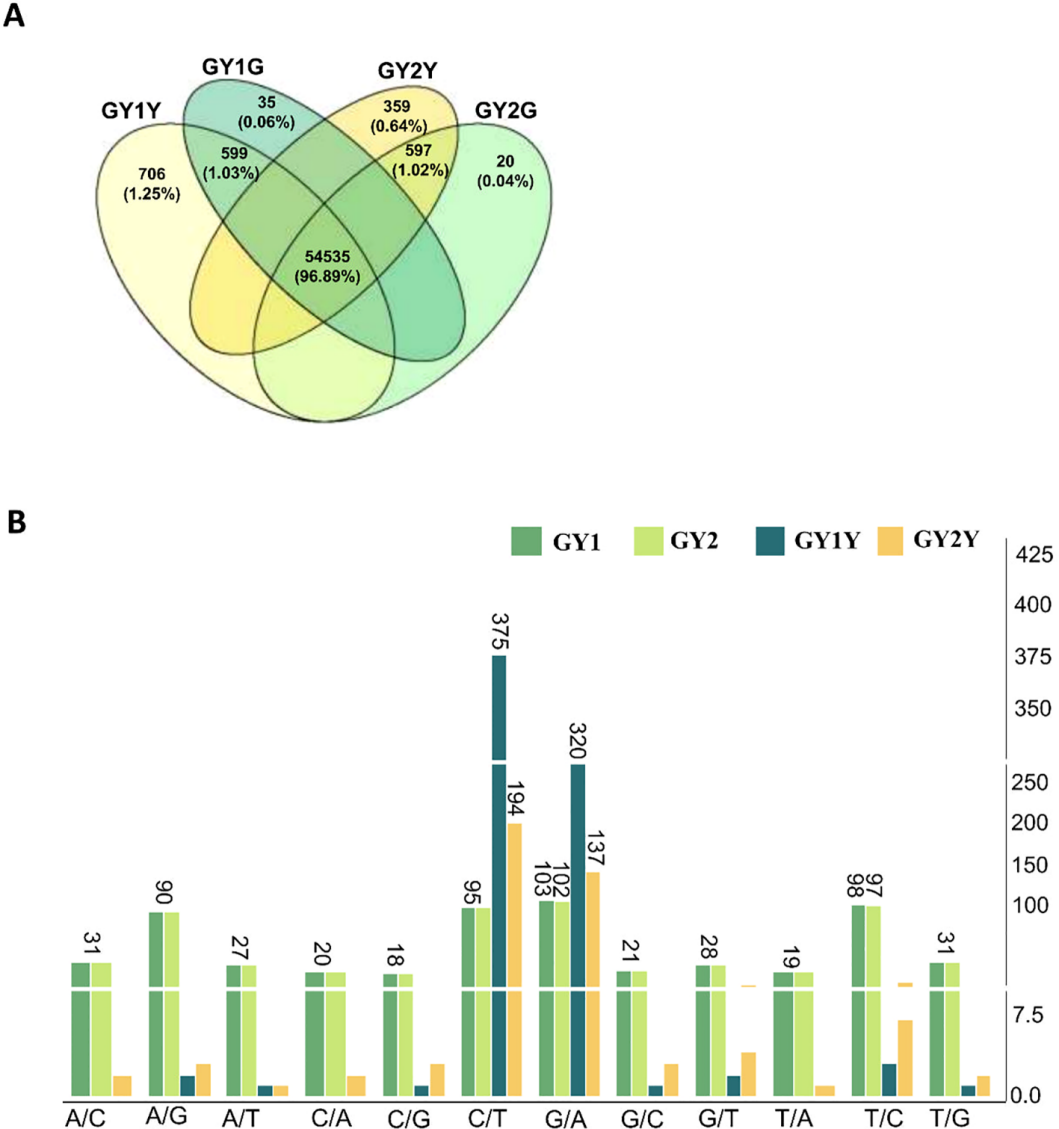
Distribution and types of SNPs caused by double EMS mutagenesis treatment. **(A)** The distribution of SNPs caused by double EMS mutagenesis treatment. **(B)** The SNP base mutation type causes double mutations; GY1Y represents the SNPs unique to the yellow leaves of GY1, GY2Y represents the SNPs unique to the yellow leaves of GY2, GY1 represents the SNPs that exists in both GY1Y and GY1G, and GY2 represents the SNPs that exist in both GY2Y and GY2G. The vertical axis represents the number of SNPs.

By contrast, the mutations induced by the second EMS application were unique to each leaf type, with 706, 35, 359, and 20 SNPs unique to GY1Y, GY1G, GY2Y, and GY2G, respectively (**Figure 4A**). The green leaves contained very few unique SNPs, suggesting that these leaves escaped the influence of the second EMS application. Therefore, the mutations responsible for the yellow leaf phenotype are likely found in genes carrying the unique SNPs induced by the second EMS treatment.

For all SNPs caused by EMS treatments, the most common mutation types were C/T and G/A, with 679 (37.8%) and 582 (36.9%) occurrences, respectively, accounting for 74.7% of the total mutations. There were 104 instances of A/G mutations, representing 7.05% of the total mutations (**Supplementary Table S4**). As shown in **Figure 4B**, the mutation benefits after twice EMS treatments are shown, among which C/T and G/A have the highest mutation benefits (**Figure 4B**). Apart from the unique SNPs in green leaves (**Supplementary Figure S3**), the distribution of mutation types was similar across the EMS-induced SNPs, indicating that the EMS mutagenesis protocol was effective.

### 3.4 Prediction of candidate genes causing leaf yellowing

Compared to the green leaves, yellow leaves had 706 and 359 unique SNPs in GY1 and GY2, respectively. The genes responsible for color differentiation are likely among these SNPs. Additionally, the genes responsible for the yellow leaf phenotype will have undergone two mutations due to the presence of sister chromatids. One mutation would be among the SNPs caused by the second EMS application and the other could be among the SNPs caused by the first or second EMS application. Eighteen genes in GY1 and three genes in GY2 met these conditions (**Supplementary Table S5**).

Functional annotation suggested that the gene responsible for the yellow leaves in GY1 is the gene encoding the eukaryotic translation initiation factor eIIso4G. A previous study demonstrated that knockout of the eIIso4G gene in Arabidopsis caused a significant decrease in chlorophyll content (Lellis et al., 2010). In GY2, the ubiquitin ligase gene *RKP* had two SNPs: one present in both the yellow and green leaves and another unique to the yellow leaves. Another study reported that a mutation in the ubiquitin ligase gene *NOT4A* can lead to a light-yellow phenotype in the leaves in Arabidopsis (Bailey et al., 2021). We speculate that the ubiquitin ligase RKP in peas may have a similar function (**Supplementary Table S5**).

## 4 Discussion

In this study, we aimed to find the transcriptional and genetic characteristic of the chimeric pea phenotype induced by EMS mutagenesis through transcriptome analysis. The experimental materials consisted of chimeric plants exhibiting both yellow and green leaves. Typically, severe yellowing in leaves leads to plant death, preventing the preservation of mutant lines for further analysis (Xu et al., 2023). In such cases, the yellow-leaved sections are unable to produce viable seeds, and the few seeds produced lack vitality and fail to germinate. Consequently, research on plants with severely yellow leaves is scarce. Through direct transcriptome analysis of the leaves of chimeric plants, we were afforded the unique opportunity to elucidate the genetic characteristic of the yellow leaf phenotype, a task previously elusive via alternative methodologies. This approach enabled the exploration of genetic disparities within a singular plant entity, thereby providing invaluable insights that would otherwise remain elusive.

Mutations from the double EMS application were effective. We used double EMS application because we wanted our material to produce two consecutive mutations. There are literatures that show that after multiple EMS mutagenesis, experimental materials will produce consecutive mutations. However, there are not many similar related articles. Therefore, we want to observe whether we can produce more interesting results by applying EMS mutagenesis multiple times. This is the first time that we have produced pea chimeras with yellow leaves after applying EMS mutagenesis twice. This is an attempt at mutagenesis. If the phenotypes produced by multiple mutagenesis are very different from those produced by only applying EMS mutagenesis once, then multiple EMS mutagenesis is also an effective means. Six chimeric leaf color mutations were found in 1,245 lines after the second EMS application, indicating that double EMS application is an effective strategy to increase the mutation probability in genes affecting leaf color. The mutation mechanism of chemical EMS treatment is relatively well understood. The N7 position of guanine (G) in DNA undergoes alkylation by the alkyl group of EMS, leading to mutations. This alkylation will cause G to no longer pair with cytosine (C) but instead with thymine (T), resulting in a purine-pyrimidine transition mutation (i.e., mutation of G/C to A/T) (Yan et al., 2021). Consistently, we found that the main mutation types that appeared after EMS application in the pea plants were C/T and G/A. The relatively low number of SNPs in the green leaves of GY1 and GY2 suggests that these tissues might have escaped the impact of EMS on mutagenesis. The EMS solution likely did not reach all of the cells in the seeds during soaking, allowing some unmutated cells to develop into green leaves. The observed heterogeneity also suggested that the seeds from the same plant mutated by EMS mutagenesis could have different genotypes and phenotypes (Chen et al., 2023). The GY1 and GY2 chimera plants likely originated from different parent plants during the first EMS application, as they exhibited different SNPs in both the yellow and green leaves.

Based on transcriptome analysis and functional annotation, the genetic mechanisms underlying the formation of yellow leaves in the GY1 and GY2 lines were hypothesized to involve the eukaryotic translation initiation factor eIFiso4G and the ubiquitin ligase RKP. The two candidate genes weren’t the structural genes relative to chlorophyll or xanthophyll biosynthesis, but they could influence the chlorophyll and xanthophyll in previous researches. In Arabidopsis, knockout of eIFiso4G led to a significant decrease in two light-harvesting complex–binding proteins, rubisco activase and carbonic anhydrase, resulting in impaired grana stacking within chloroplasts (Dufil et al., 2022). This could explain the enrichment of DEGs related to chloroplast development and function found in the GO enrichment analysis of GY1 (**Supplementary Table S6**). Other enriched GO terms included response to stimulus and stress (**Supplementary Table S6**). The knockout lines showed effects on electron absorbance, trapping, and electron transport, which acted as stimuli for the plant cells (Dufil et al., 2022).

Ubiquitin ligase has been shown to regulate the expression of proteins containing a pentatricopeptide repeat domain (Wang et al., 2021), which is crucial for stabilizing gene transcripts, promoting post-transcriptional processing, and facilitating protein translation in ribosomes by binding to organelle RNA (Rojas et al., 2018). Indeed, enriched GO terms related to ribosome development were observed in the chimeric pea plants. Abnormal cytosolic ribosomes can impact chloroplast development (Wang et al., 2017), leading to stress responses, and the abnormal chloroplasts will lead to a decreased chlorophyll content. The blockage of chlorophyll biosynthesis leads to the accumulation of 5-aminolevulinic acid, a precursor of chlorophyll, which disrupts other amino acid biosynthesis pathways (Ilag et al., 1994). Consistently, we found that the enriched KEGG pathways in the yellow leaves of the chimera pea plants were mainly related to amino acid biosynthesis in both GY1 and GY2 (**Supplementary Table S7**). The genetic underpinnings governing the manifestation of yellow leaves in GY1 and GY2 are presumed to be distinct, as evidenced by their disparate profiles of DEGs, enriched GO terms, KEGG pathways, and EMS-induced SNPs. Nonetheless, the shared manifestation of the yellow leaf phenotype in both lines suggests a degree of commonality in their respective suites of DEGs implicated in this characteristic trait.

In the fields of plant breeding and horticulture, chimera peas exhibiting yellow leaf characteristics have demonstrated remarkable research value and extensive application potential. From a plant breeding perspective, these peas, as exceptional genetic materials, offer an ideal experimental paradigm for investigating the phytochrome synthesis pathway and its underlying gene regulatory mechanisms (Tsyganov et al., 2007). Their genetic traits profoundly illuminate the intricate and intricate pigment synthesis network within plants, furnishing breeders with abundant genetic resources and a robust theoretical framework. By integrating modern biotechnologies (e.g., gene editing, molecular marker-assisted selection) with traditional breeding methodologies, scientists can precisely manipulate the pigment synthesis pathway in peas, thereby enabling the targeted breeding of novel varieties characterized by unique coloration, enhanced environmental adaptability, and stress resistance (Hasan et al., 2021). The successful cultivation of these novel varieties not only significantly enriches the genetic diversity of peas but also lays a solid genetic foundation for the sustainability and resilience of agricultural production. And in the realm of horticulture, the yellow-leaved chimera peas stand out as a focal point in landscape design due to their distinctive visual appeal (Lakitan et al., 2023). Their vivid yellow leaves create a stark contrast amidst the verdant plantscape, imbuing horticultural creations with unparalleled artistic allure and visual dynamism. These peas hold vast potential for application in diverse settings, including home gardens, public green spaces, and theme parks, where they can markedly elevate the ornamental value and aesthetic appeal of landscapes. Furthermore, the introduction of yellow-leaved peas has spurred the diversification and innovative evolution of horticultural plant varieties, catering to the market’s pressing demand for novel and aesthetically pleasing horticultural products, thereby driving technological advancements and industrial upgrading within the horticultural sector.

## Data Availability

The datasets presented in this study can be found in online repositories. The names of the repository/repositories and accession number(s) can be found below: https://ngdc.cncb.ac.cn/gsub/submit/bioproject/subPRO038859, PRJCA026213.
