# Supplementary material for: Transcriptional and genetic characteristic of chimera pea generation via double ethyl methanesulfonate-induced mutation revealed by transcription analysis
Source: Front Plant Sci. 2024 Oct 1;15:1439547. doi: 10.3389/fpls.2024.1439547 (PMC11473339; doi:10.3389/fpls.2024.1439547)
Supplement: Supplementary file 1 [file DataSheet1.docx]

Supplementary Material


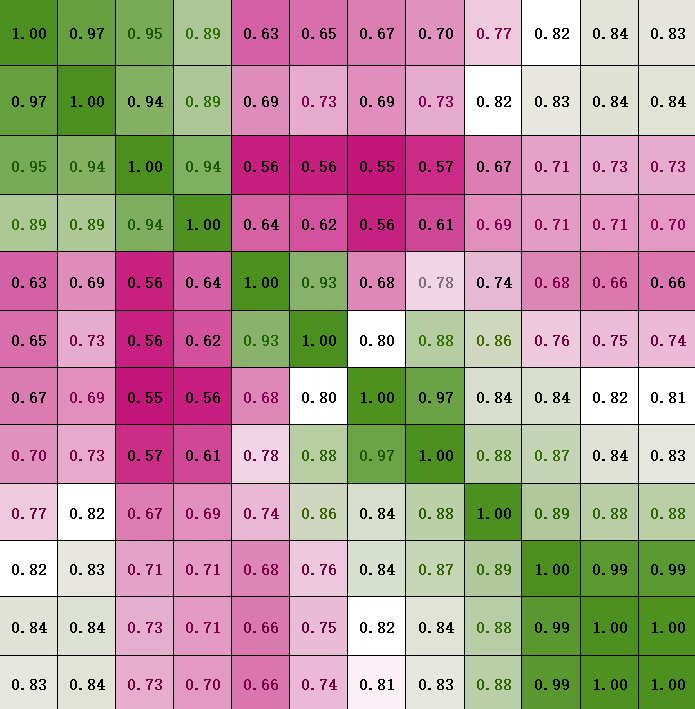

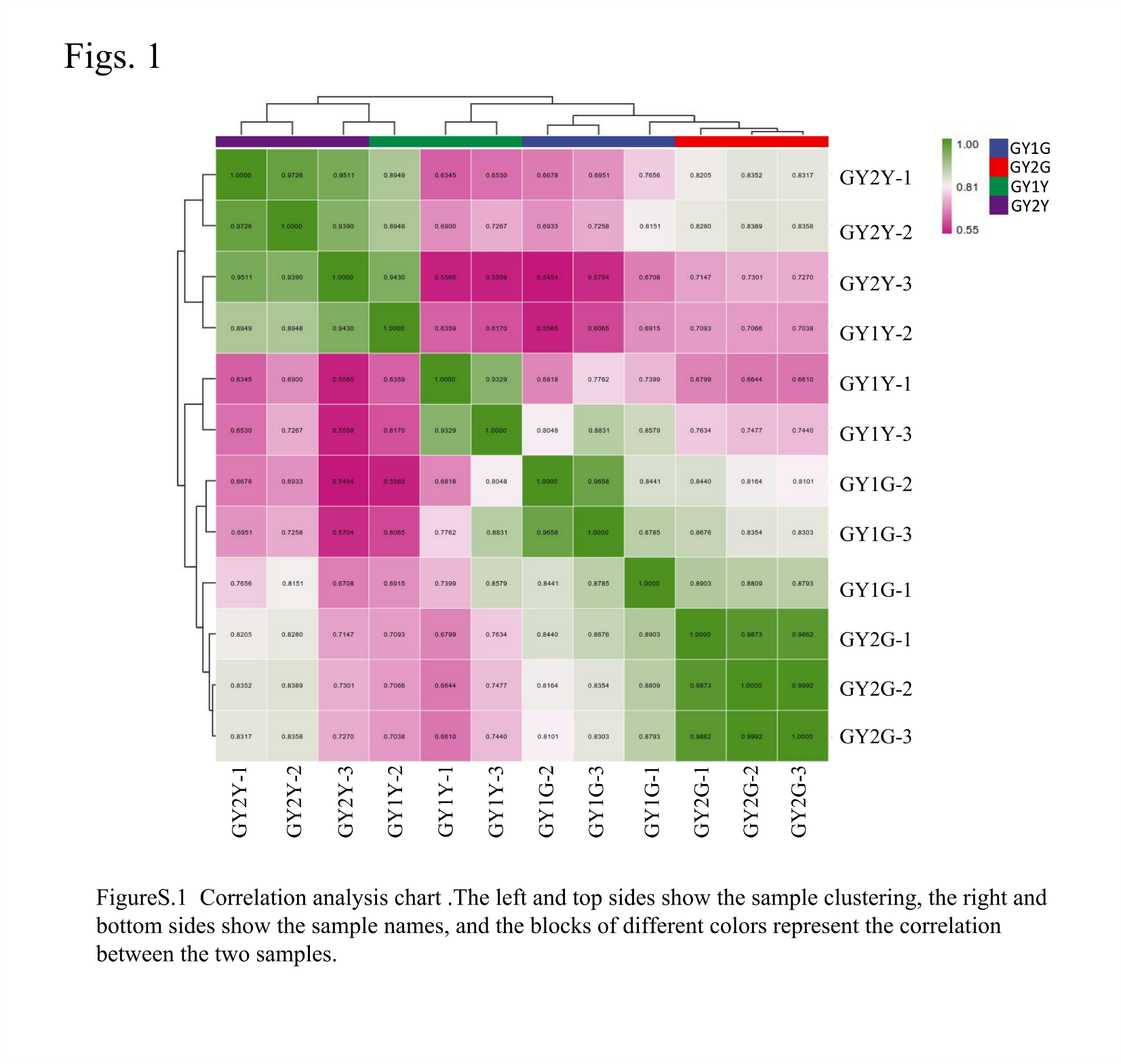


Figure S1. Correlation analysis of the differentlly expression genes in GY1G, GY1Y, GY2G and GY2Y. The left and top sides show the sample clustering, the right and bottom sides show the sample names, and the blocks of different colors represent the correlation between the two samples.


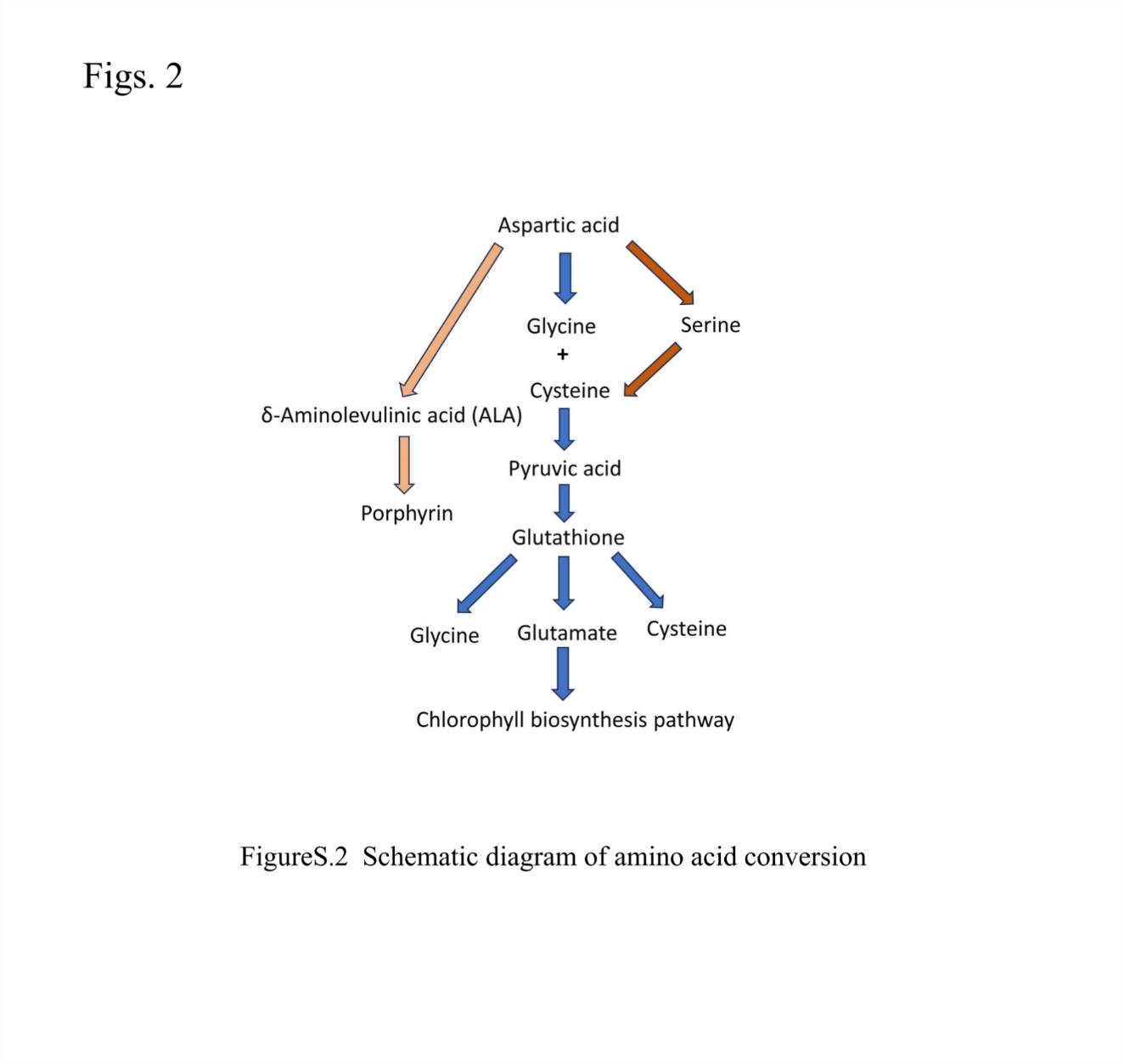


Figure S2. The relationship of the amino acid biosynthesis and the chlorophyll biosynthesis


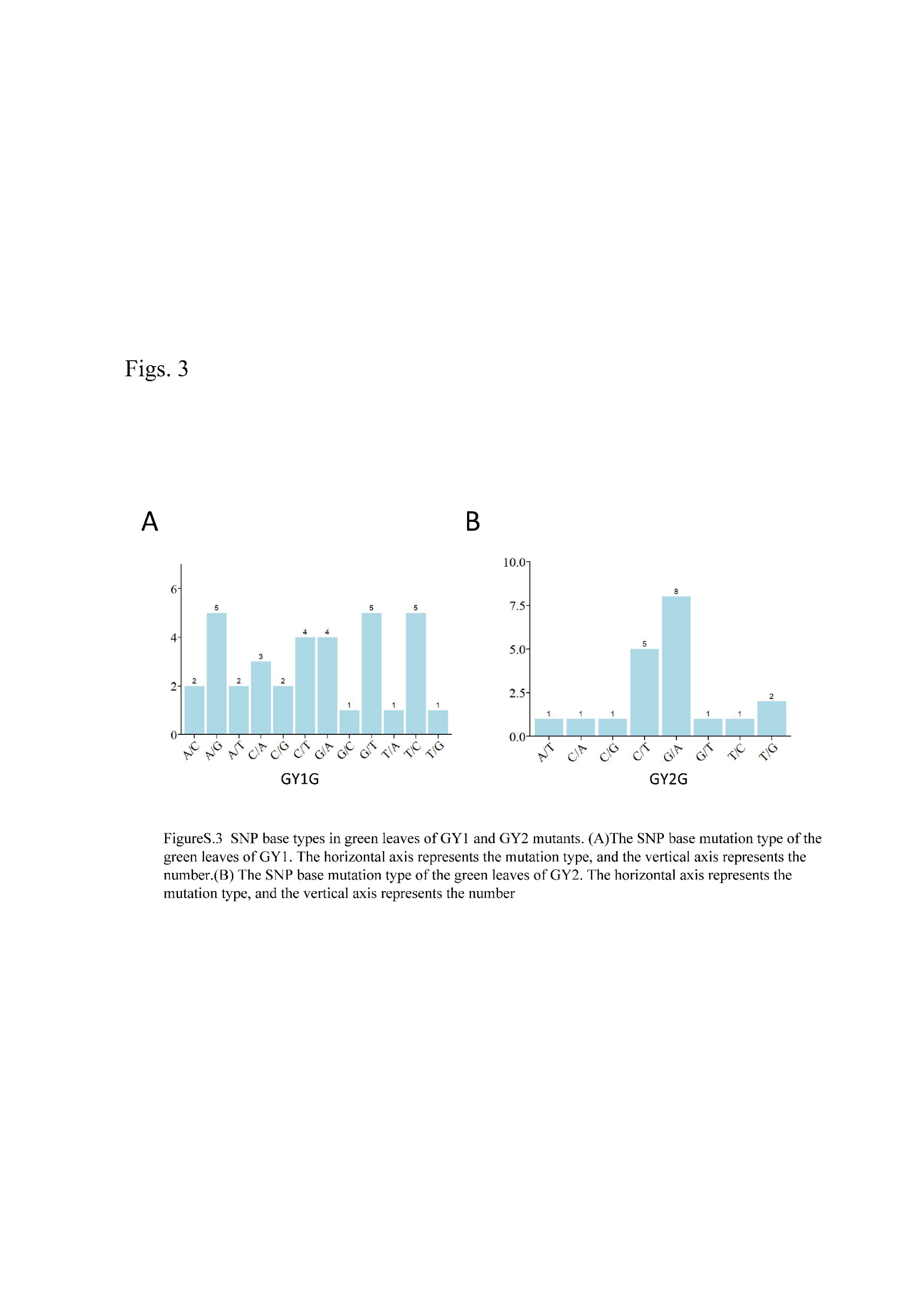


Figure S3. The distribution of the SNP base types in green leaves of GY1 and GY2. (A) The SNP base mutation type of the green leaves of GY1. The horizontal axis represents the mutation type, and the vertical axis represents the number. (B) The SNP base mutation type of the green leaves of GY2. The horizontal axis represents the mutation type, and the vertical axis represents the number

Table S1. The chlorophyll content of GY1Y, GY1G, GY2Y and GY2G

|  | GY1Y | GY1G | GY2Y | GY2G |
| --- | --- | --- | --- | --- |
| Total chlorophyll content(mg/g) | 0.06 | 0.72 | 0.05 | 0.80 |
| Chlb(mg/g) | 0.02 | 0.40 | 0.02 | 0.45 |
| Chla(mg/g) | 0.04 | 0.32 | 0.04 | 0.35 |

Table S2. The lutein content of GY1Y, GY1G, GY2Y and GY2G

| sample | GY1Y | GY1G | GY2Y | GY2G |
| --- | --- | --- | --- | --- |
| Lutein content(μg/100g) | 436 | 2.17 | 450 | 1.76 |

Table S3. The lists of the raw sequencing data

| Type | GY1G | GY1Y | GY2G | GY2Y |
| --- | --- | --- | --- | --- |
| Total raw reads | 40374897 | 41099278 | 50287855 | 43067837 |
| Total clean reads | 39674053 | 40601464 | 49513111 | 42406297 |
| Total clean bases (bp) | 5709533557 | 6116613873 | 7458123038 | 6389785550 |
| Q20 percentage (%) | 97.93 | 97.98 | 97.85 | 97.92 |
| Q30 percentage (%) | 95.94 | 96.08 | 96.05 | 95.92 |

| Type | all snps | GY1 | GY1Y | GY1G | GY2 | GY2Y | GY2G |
| --- | --- | --- | --- | --- | --- | --- | --- |
| A/G | 104 | 90 | 2 | 5 | 90 | 3 | 0 |
| A/T | 32 | 27 | 1 | 2 | 27 | 1 | 1 |
| A/C | 36 | 31 | 0 | 2 | 31 | 2 | 0 |
| C/G | 25 | 18 | 1 | 2 | 18 | 3 | 1 |
| C/T | 678 | 95 | 375 | 4 | 95 | 194 | 5 |
| C/A | 26 | 20 | 0 | 3 | 20 | 2 | 1 |
| G/A | 582 | 103 | 320 | 4 | 102 | 137 | 8 |
| G/C | 26 | 21 | 1 | 1 | 21 | 3 | 0 |
| G/T | 43 | 28 | 2 | 5 | 28 | 4 | 1 |
| T/A | 0 | 19 | 0 | 1 | 19 | 1 | 0 |
| T/C | 117 | 98 | 3 | 5 | 98 | 7 | 1 |
| T/G | 40 | 31 | 1 | 1 | 31 | 2 | 2 |

Table S4. The statistics of SNP

Note.all snps in the table header represents all SNPs generated after two mutagenesis procedures. GY1Y represents the SNPs unique to the yellow leaves of GY1, GY2Y represents the SNPs unique to the yellow leaves of GY2, GY1 represents the SNPs that exists in both GY1Y and GY1G, and GY2 represents the SNPs that exist in both GY2Y and GY2G.

Table S5. List of the genes carried two SNPs in GY1 and GY2

| Sample | GeneID | Chr | Position | type | Element | Position | type | Element | Function |
| --- | --- | --- | --- | --- | --- | --- | --- | --- | --- |
| GY1 | LOC127073456 | NC_066582.1 | 70723827 | G/A | exonic | 70726108 | G/A | exonic | large proline-rich protein BAT3 |
|  | LOC127078917 | NC_066583.1 | 92737753 | C/T | exonic | 92737712 | G/A | exonic | lariat debranching enzyme |
|  | LOC127082772 | NC_066579.1 | 449059033 | C/G | exonic | 449058670 | G/C | exonic | ribosomal protein SA |
|  | LOC127084612 | NC_066583.1 | 61439262 | C/T | exonic | 352588169 | C/T | exonic | lysosomal beta glucosidase-like protein |
|  | LOC127086143 | NC_066579.1 | 462790714 | C/T | exonic | 462788016 | C/T | exonic | elongation factor 1-gamma |
|  | LOC127086463 | NC_066583.1 | 2526446 | C/T | exonic | 2526444 | G/A | exonic | EF1Bgamma3 |
|  | LOC127092766 | NC_066584.1 | 110155394 | G/A | exonic | 110151805 | G/A | exonic | Cysteine β lyase |
|  | LOC127092779 | NC_066584.1 | 239249988 | G/A | exonic | 239248711 | G/A | exonic | arginine/serine-rich coiled-coil protein 2 isoform X1 |
|  | LOC127098276 | NC_066584.1 | 237285984 | G/A | exonic | 237280476 | C/T | exonic | Serine/Threonine Kinase Proteins |
|  | LOC127100400 | NC_066585.1 | 210859160 | G/A | exonic | 210857021 | G/A | exonic | Bromo adjacent-like domain（BRAD） |
|  | LOC127100446 | NC_066585.1 | 17014262 | G/A | exonic | 17012240 | G/A | exonic | The exocyst complex |
|  | LOC127107210 | NC_066585.1 | 541741308 | C/T | exonic | 541740033 | G/A | exonic | ABC transporter E family member 2-like protein |
|  | LOC127108465 | NC_066585.1 | 16349260 | C/T | exonic | 16349230 | C/T | exonic | Mitochondrial nudix hydrolase 18 |
|  | LOC127117500 | NC_066580.1 | 3242359 | C/T | intronic | 3240270 | G/A | exonic | heterogeneous nuclear ribonucleoprotein 1 |
|  | LOC127117907 | NC_066580.1 | 36047572 | C/T | exonic | 36047058 | C/T | exonic | la-related protein 1A |
|  | LOC127132518 | NC_066581.1 | 58535577 | C/T | exonic | 58535432 | C/T | exonic | Cysteine protease |
|  | LOC127138454 | NC_066582.1 | 17007658 | G/T | exonic | 17006298 | G/A | exonic | eukaryotic translation initiation factor iso4G |
| GY2 | LOC127074875 | NC_066582.1 | 247705062 | G/A | exonic | 247709840 | A/G | exonic | Arp2/3 complex 34 kDa subunit |
|  | LOC127074969 | NC_066582.1 | 258803039 | A/T | exonic | 258802050 | A/T | exonic | nicotinamidase 1 isoform X2 |
|  | LOC127096605 | NC_066584.1 | 119401137 | G/A | exonic | 119400741 | G/A | ncRNA_exonic | ubiquitin ligase RKP |

Notes. The first 'position' in the table header represents the SNP mutation caused by the first mutagenesis procedure, and the second 'position' represents the SNP mutation caused by the second mutagenesis procedure.

Table S6. GO enrichment of differentlly expression genes in GY1 and GY2.

|  | Category | Term | Up | Down | Degs | Total | Pvalue |
| --- | --- | --- | --- | --- | --- | --- | --- |
| GY1 | BP | response to chitin | 42 | 5 | 47 | 175 | 4.26E-07 |
|  | BP | response to stimulus | 494 | 379 | 873 | 6062 | 5.74E-07 |
|  | BP | response to oxygen-containing compound | 200 | 116 | 316 | 1934 | 7.56E-07 |
|  | CC | chloroplast | 254 | 76 | 330 | 2085 | 1.21E-06 |
|  | CC | chloroplast stroma | 111 | 20 | 131 | 719 | 5.00E-06 |
|  | CC | plastid stroma | 112 | 20 | 132 | 728 | 5.92E-06 |
|  | CC | plastid | 261 | 77 | 338 | 2182 | 6.70E-06 |
|  | BP | response to stress | 309 | 222 | 531 | 3553 | 8.63E-06 |
|  | BP | defense response | 126 | 68 | 194 | 1137 | 1.03E-05 |
|  | BP | response to wounding | 38 | 26 | 64 | 297 | 1.65E-05 |
|  | BP | trehalose biosynthetic process | 6 | 4 | 10 | 19 | 3.61E-05 |
|  | BP | protein targeting to chloroplast | 14 | 2 | 16 | 44 | 6.07E-05 |
|  | BP | Establishment of protein localization to chloroplast | 14 | 2 | 16 | 44 | 6.07E-05 |
|  | BP | response to oxidative stress | 67 | 22 | 89 | 466 | 6.12E-05 |
|  | BP | response to hormone | 173 | 114 | 287 | 1826 | 6.18E-05 |
|  | BP | trehalose metabolic process | 6 | 4 | 10 | 20 | 6.39E-05 |
|  | BP | response to acid chemical | 131 | 79 | 210 | 1284 | 6.87E-05 |
|  | BP | ethylene metabolic process | 7 | 4 | 11 | 24 | 7.54E-05 |
|  | BP | ethylene biosynthetic process | 7 | 4 | 11 | 24 | 7.54E-05 |
|  | BP | cellular alkene metabolic process | 7 | 4 | 11 | 24 | 7.54E-05 |
| GY2 | CC | cytosolic ribosome | 122 | 9 | 131 | 270 | 1.52E-28 |
|  | CC | ribosomal subunit | 114 | 3 | 117 | 232 | 1.65E-27 |
|  | CC | ribosome | 130 | 10 | 140 | 311 | 2.85E-26 |
|  | MF | structural constituent of ribosome | 118 | 2 | 120 | 252 | 3.76E-24 |
|  | CC | polysomal ribosome | 67 | 0 | 67 | 114 | 4.80E-21 |
|  | CC | cytosolic large ribosomal subunit | 62 | 1 | 63 | 109 | 2.39E-19 |
|  | CC | large ribosomal subunit | 67 | 1 | 68 | 124 | 4.56E-19 |
|  | MF | structural molecule activity | 121 | 7 | 128 | 328 | 1.38E-16 |
|  | CC | polysome | 67 | 1 | 68 | 136 | 2.79E-16 |
|  | CC | cytosolic small ribosomal subunit | 44 | 1 | 45 | 95 | 3.01E-10 |
|  | CC | small ribosomal subunit | 47 | 2 | 49 | 108 | 3.29E-10 |
|  | CC | proteasome core complex | 19 | 0 | 19 | 25 | 1.02E-09 |
|  | CC | proteasome complex | 34 | 0 | 34 | 69 | 1.25E-08 |
|  | BP | response to stimulus | 609 | 701 | 1310 | 6062 | 3.23E-08 |
|  | MF | oxidoreductase activity | 112 | 129 | 241 | 906 | 6.44E-08 |
|  | BP | response to abiotic stimulus | 266 | 289 | 555 | 2360 | 8.43E-08 |
|  | BP | cytoplasmic translation | 34 | 2 | 36 | 78 | 8.68E-08 |
|  | CC | endopeptidase complex | 34 | 0 | 34 | 74 | 1.13E-07 |
|  | BP | carbohydrate biosynthetic process | 34 | 54 | 88 | 268 | 1.43E-07 |
|  | BP | translation | 131 | 9 | 140 | 483 | 2.45E-07 |

Table S7. KEGG pathway enrichment of differentlly expression genes in GY1 and GY2

|  | Pathway | Level | Up | Down | DEG | Total | Pvalue |
| --- | --- | --- | --- | --- | --- | --- | --- |
| GY1 | Valine, leucine and isoleucine biosynthesis | Metabolism | 8 | 0 | 8 | 19 | 0.00114 |
|  | Aminoacyl-tRNA biosynthesis | Genetic Information Processing | 15 | 1 | 16 | 63 | 0.003386 |
|  | Starch and sucrose metabolism | Metabolism | 20 | 25 | 45 | 252 | 0.006313 |
|  | Cysteine and methionine metabolism | Metabolism | 20 | 6 | 26 | 128 | 0.006715 |
|  | Arginine and proline metabolism | Metabolism | 13 | 6 | 19 | 87 | 0.008797 |
|  | Plant-pathogen interaction | Organismal Systems | 33 | 8 | 41 | 231 | 0.009868 |
|  | Glycine, serine and threonine metabolism | Metabolism | 16 | 4 | 20 | 96 | 0.012368 |
|  | Alanine, aspartate and glutamate metabolism | Metabolism | 12 | 4 | 16 | 73 | 0.014963 |
|  | Ribosome | Genetic Information Processing | 63 | 0 | 63 | 396 | 0.0189 |
|  | Pantothenate and CoA biosynthesis | Metabolism | 10 | 5 | 15 | 69 | 0.019482 |
|  | Arginine biosynthesis | Metabolism | 12 | 1 | 13 | 59 | 0.025686 |
|  | Protein processing in endoplasmic reticulum | Genetic Information Processing | 44 | 7 | 51 | 321 | 0.033281 |
|  | Porphyrin metabolism | Metabolism | 9 | 2 | 11 | 49 | 0.033894 |
|  | Ascorbate and aldarate metabolism | Metabolism | 8 | 5 | 13 | 62 | 0.03732 |
|  | Ribosome biogenesis in eukaryotes | Genetic Information Processing | 17 | 2 | 19 | 103 | 0.04663 |
|  | Sulfur metabolism | Metabolism | 6 | 3 | 9 | 43 | 0.076416 |
|  | Vitamin B6 metabolism | Metabolism | 4 | 2 | 6 | 26 | 0.092974 |
|  | Lysine biosynthesis | Metabolism | 3 | 1 | 4 | 16 | 0.126216 |
|  | beta-Alanine metabolism | Metabolism | 6 | 7 | 13 | 76 | 0.139591 |
|  | Valine, leucine and isoleucine degradation | Metabolism | 8 | 3 | 11 | 63 | 0.148981 |
| GY2 | Ribosome | Genetic Information Processing | 184 | 1 | 185 | 396 | 1.26E-32 |
|  | Proteasome | Genetic Information Processing | 37 | 0 | 37 | 72 | 9.17E-09 |
|  | Glycine, serine and threonine metabolism | Metabolism | 21 | 13 | 34 | 96 | 0.000691 |
|  | Diterpenoid biosynthesis | Metabolism | 4 | 9 | 13 | 30 | 0.004649 |
|  | Alanine, aspartate and glutamate metabolism | Metabolism | 16 | 9 | 25 | 73 | 0.005593 |
|  | Brassinosteroid biosynthesis | Metabolism | 2 | 6 | 8 | 15 | 0.005664 |
|  | Tyrosine metabolism | Metabolism | 9 | 8 | 17 | 45 | 0.007041 |
|  | Inositol phosphate metabolism | Metabolism | 12 | 11 | 23 | 73 | 0.022077 |
|  | Plant-pathogen interaction | Organismal Systems | 30 | 31 | 61 | 231 | 0.025185 |
|  | Valine, leucine and isoleucine degradation | Metabolism | 17 | 3 | 20 | 63 | 0.029043 |
|  | Fatty acid degradation | Metabolism | 11 | 12 | 23 | 75 | 0.030165 |
|  | Propanoate metabolism | Metabolism | 10 | 4 | 14 | 44 | 0.060534 |
|  | Glyoxylate and dicarboxylate metabolism | Metabolism | 10 | 20 | 30 | 111 | 0.073675 |
|  | Valine, leucine and isoleucine biosynthesis | Metabolism | 7 | 0 | 7 | 19 | 0.083027 |
|  | Nitrogen metabolism | Metabolism | 5 | 7 | 12 | 39 | 0.097802 |
|  | Flavone and flavonol biosynthesis | Metabolism | 0 | 2 | 2 | 3 | 0.113204 |
|  | Monobactam biosynthesis | Metabolism | 2 | 3 | 5 | 13 | 0.116034 |
|  | Isoquinoline alkaloid biosynthesis | Metabolism | 4 | 2 | 6 | 17 | 0.125756 |
|  | Butanoate metabolism | Metabolism | 4 | 3 | 7 | 21 | 0.131114 |
|  | Glycolysis / Gluconeogenesis | Metabolism | 22 | 20 | 42 | 173 | 0.159263 |
